# Association of XIST/miRNA155/Gab2/TAK1 cascade with the pathogenesis of anti-phospholipid syndrome and its effect on cell adhesion molecules and inflammatory mediators

**DOI:** 10.1038/s41598-023-45214-z

**Published:** 2023-11-01

**Authors:** Mai A. Abd-Elmawla, Yumn A. Elsabagh, Nora M. Aborehab

**Affiliations:** 1 Biochemistry Department, Faculty of Pharmacy, Cairo University, Cairo, 11562 Egypt; 2 Internal Medicine Department (Rheumatology and Clinical Immunology Unit), Faculty of Medicine, Cairo University, Cairo, Egypt; 3 Biochemistry Department, Faculty of Pharmacy, October University for Modern Sciences and Arts (MSA), Giza, 12451 Egypt

**Keywords:** Biochemistry, Immunology, Molecular biology

## Abstract

Anti-phospholipid syndrome (APS) is an autoimmune disease characterized by thrombosis and miscarriage events. Still, the molecular mechanisms underlying APS, which predisposes to a wide spectrum of complications, are being explored. Seventy patients with primary and secondary APS were recruited, in addition to 35 healthy subjects. Among APS groups, the gene expression levels of XIST, Gab2, and TAK1 were higher along with declined miRNA155 level compared with controls. Moreover, the sera levels of ICAM-1, VCAM-1, IL-1ꞵ, and TNF-α were highly elevated among APS groups either primary or secondary compared with controls. The lncRNA XIST was directly correlated with Gab2, TAK1, VCAM-1, ICAM-1, IL-1ꞵ, and TNF-α. The miRNA155 was inversely correlated with XIST, Gab2, and TAK1. Moreover, ROC curve analyses subscribed the predictive power of the lncRNA XIST and miRNA155, to differentiate between primary and secondary APS from control subjects. The lncRNA XIST and miRNA155 are the upstream regulators of the Gab2/TAK1 axis among APS patients via influencing the levels of VCAM-1, ICAM-1, IL1ꞵ, and TNF-α which propagates further inflammatory and immunological streams. Interestingly, the study addressed that XIST and miRNA155 may be responsible for the thrombotic and miscarriage events associated with APS and provides new noninvasive molecular biomarkers for diagnosing the disease and tracking its progression.

## Introduction

Anti-phospholipid syndrome (APS) is an autoimmune disease characterized by thrombosis together with miscarriage events as a consequence of APS antibodies, such as lupus anticoagulant, anti-cardiolipin and antiβ2-glycoprotein antibodies^1^. APS can develop as a primary disease or secondary to another disease, such as systemic lupus erythematosus (SLE)^2,3^. Although APS antibodies predispose to thrombotic events and obstetrical complications, also other aspects may play a part such as environmental factors, inflammatory factors, or other non-immunological pro-coagulant mediators^4^.

Nowadays, noncoding RNAs like microRNAs (miRNAs) and long non-coding RNAs (lncRNAs), are key players in the regulation of a battery of genes^5^. The lncRNA X-inactive specific transcript (XIST) is located in the X chromosome inactivation center of chromosome Xq13.2. and acts as a crucial director in cell proliferation and differentiation^6^. XIST also participates in the mechanism of several detrimental manifestations for instance obstetrical complications and thrombosis^7,8^. Earlier literature addressed the chief role of the lncRNA XIST in triggering inflammation and innate immunity, where it propagates cascades of inflammatory cytokines such as tumor necrosis factor (TNF)-α, interleukin (IL)-1β, and IL−6 leading to further immunological stimulation^7,9^. However, the precise role of XIST in sex-biased diseases such as APS remains elusive.

Interestingly, it was found that XIST mediates its inflammatory role via participating in various signaling pathways such as the Grb-associated binding (Gab) inflammatory pathway. Gab proteins are a cluster of intracellular signaling molecules, comprising Gab1, Gab2, and Gab3^10,11^. Particularly, Gab2 activates key signaling proteins in the inflammatory signaling pathways such as transforming growth factor beta-activated kinase 1 (TAK1) which stimulates the expression of cell adhesion molecules (CAMs) and cytokines release. Moreover, Gab2 silencing in endothelial cells declines the levels of TNFα, IL-1β, and CAMs^10^. Consequentially, the ongoing inflammation and thrombotic milieu encourage the adhesiveness of the endothelial cells, platelets, and leucocytes leading to a pro-coagulant tendency and a diminution of endometrial blood flow, thus raising the possibility of miscarriages; which one of the harmful complications of APS^12^.

Indeed, most lncRNAs including XIST exert their role in various signaling pathways via interaction with miRNAs to control downstream targets^6,13^. Based on bioinformatics tools to explore lncRNA-miRNA interactions, miRNA155 (ENSG00000283904) was nominated as one of the potential downstream targets of XIST. Lin et al, reported the link between XIST and miRNA155, whereas the overexpressed XIST was able to partially control the protein expression levels of some genes previously recognized as direct targets of miRNA155^14^. However, their precise roles were not recognized in APS.

Collectively, the target of the current research was intended to investigate the molecular mechanisms of lncRNA XIST and miRNA155 among APS patients either primary or secondary. Specifically, the impact of these non-coding RNAs on the inflammatory Gab2/TAK1 axis and their downstream targets vascular cell adhesion molecules (VCAM-1) and intracellular cell adhesion molecules (ICAM-1), IL-1ꞵ, and TNF-α). Additionally, the study was aiming to highlight their associated role in the diagnosis and prognosis of APS.

## Results

### Patient’s characteristics

The study includes primary and secondary APS groups, in addition to control subjects. The three groups were comparable in terms of age, sex, and weight. The average disease duration of patients enrolled in our study was 20.4 months for those with primary APS and 26.8 months for those with secondary APS. Both groups did not differ significantly concerning the duration of the disease. The clinical symptoms of APS patients are shown in Supplementary Table S1.

### Expression of XIST and miRNA155

**Figure 1:**
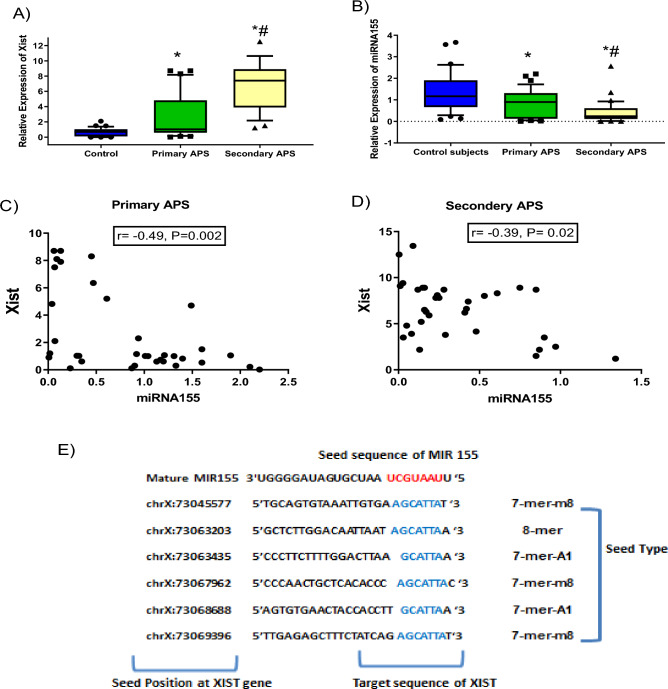
Comparison of gene expression profiles of lncRNA XIST (**A**) and miRNA155 (**B**) between healthy subjects, primary and secondary APS patients. Spearman correlation between XIST and miRNA155 among primary (**C**) and secondary (**D**) APS patients. (**E**) Putative binding sites were detected between XIST (target sequences labeled with blue) and miRNA155 (at seed sequence labeled with red) using a transcriptome-wide microRNA target prediction tool (www.mircode.org). (**A**–**B**)The box represents the 25–75% percentiles; the line inside the box represents the median and the error bars lines represent the 10–90% percentiles. * Significance difference with the control group. # Significance difference with primary APS.

The expression level of XIST was increased in primary and secondary APS patients compared with controls to a median of 1.6-fold (*P *= 0.01) and 10.5-fold (*P *< 0.001), respectively. Moreover, XIST expression was dramatically elevated among secondary APS compared with primary ones (*P* < 0.001). Contrariwise, miRNA155 was significantly decreased among primary and secondary APS patients compared with controls to a median of 0.7-fold (*P * = 0.04) and 0.3-fold (*P * < 0.001), respectively, as shown in Fig. 1A,B. Additionally, the current study recorded a marked decrease in miRNA155 expression among secondary patients compared to primary ones (*P * = 0.02). Importantly, the expression level of XIST and miRNA155 showed an inverse correlation among primary and secondary APS patients (r = − 0.49, *P *= 0.002 & r = − 0.39, *P *= 0.02, respectively) as shown in Fig. 1C,D. Using bioinformatics tools as further validation for the relationship between XIST and miRNA155, there were six base pairing positions at the lncRNA XIST (target sequences labeled with blue) and mature miRNA155 at the seed sequence (labeled with red) as shown in Fig. 1E.

### Expression of Gab2 and TAK1.

**Figure 2:**
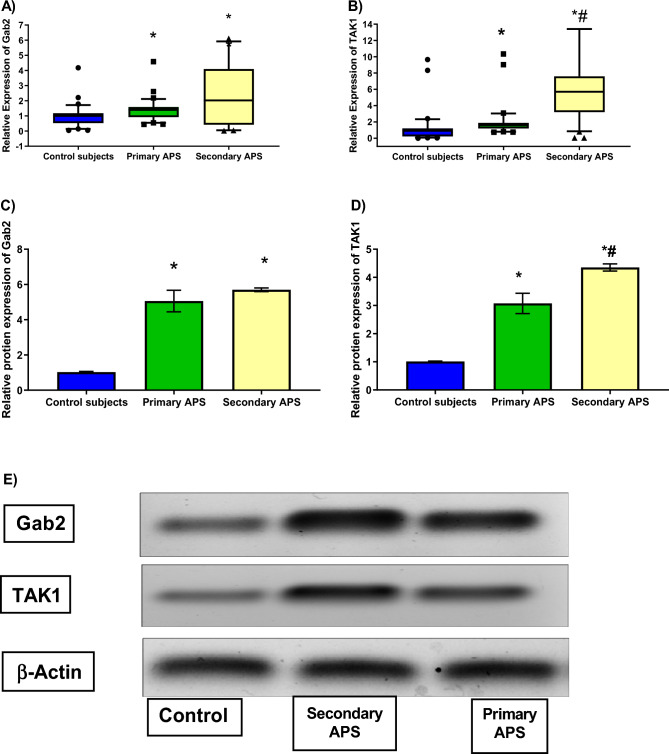
Comparison of the relative gene expression of Gab2 (**A**) and TAK1 (**B**) as well as relative protein expression of Gab2 (**C**) and TAK1 (**D**) between healthy subjects, primary and secondary APS groups. (**A**–**B**) The box represents the 25–75% percentiles; the line inside the box represents the median and the error bars represent the 10–90% percentiles. (**C**–**D**) Data are represented as mean ± SD. (**E**) Representative blots of Gab2 and TAK1. ꞵ actin was used as the loading control. Data are represented as mean ± SD. Gab2: Grb2-associated binding, TAK1: Transforming growth factor beta-activated kinase 1. * Significance difference with the control group. # Significance difference with primary APS.

As shown in Fig. 2A,B, the gene expression level of Gab2 was significantly higher among primary and secondary APS patients as compared with controls to 1.4-fold (*P *= 0.04) and 2-fold (*P* = 0.002), respectively. But no changes were observed within APS groups. Moreover, the gene expression of TAK1 was markedly higher among primary and secondary APS patients compared with healthy cases to 1.7-fold (*P *= 0.03) and 6.1-fold (*P *< 0.001), respectively. Within APS patients, TAK1 was markedly higher among secondary patients compared to primary ones (*P *= 0.0004). For more verification, the protein expression levels of both Gab2 and TAK1 were investigated. As presented in Fig. 2C,E, the protein expression of Gab2 and TAK1 were higher among primary and secondary APS patients compared with controls. Again, no changes were observed between primary and secondary APS groups in terms of Gab2, while TAK1 was significantly higher among secondary patients.

### Cell adhesion molecules & cytokines levels

**Figure 3:**
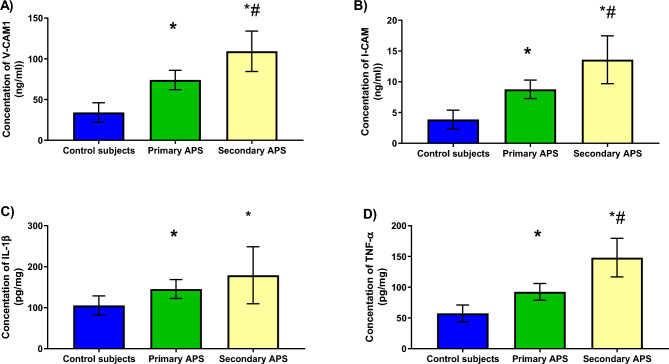
Serum levels of VCAM-1 (**A**), ICAM-1 (**B**), IL-1ꞵ (**C**), and TNF-α (**D**) among healthy subjects, primary and secondary APS groups. Data are represented as mean ± SD. ICAM-11: Intracellular cell adhesion molecules, IL-1ꞵ: Interleukin1ꞵ, TNF-α: Tumor necrosis factor-α, VCAM-1: Vascular cell adhesion molecules. *Significance difference with the control group. # Significance difference with primary APS.

Cell adhesion molecules were significantly higher among APS patients. The sera level of VCAM-1 was highly elevated among primary and secondary APS patients to 2.3-fold (*P *< 0.0001) and 3.4-fold (*P *< 0.0001), respectively, compared to healthy subjects. Comparably, the sera level of VCAM-1 was significantly elevated among secondary patients in comparison with primary ones (*P *= 0.0003). By the same token, the sera level of ICAM-1 was highly elevated among primary and secondary APS patients to 2.2-fold (*P *< 0.0001) and 3.5-fold (*P *< 0.0001), respectively, compared to healthy subjects. The sera level of ICAM-1 was significantly higher among secondary patients compared to primary ones (*P *= 0.001) as shown in Fig. 3.

### Correlation of XIST & miRNA155 with the other studied parameters

**Figure 4:**
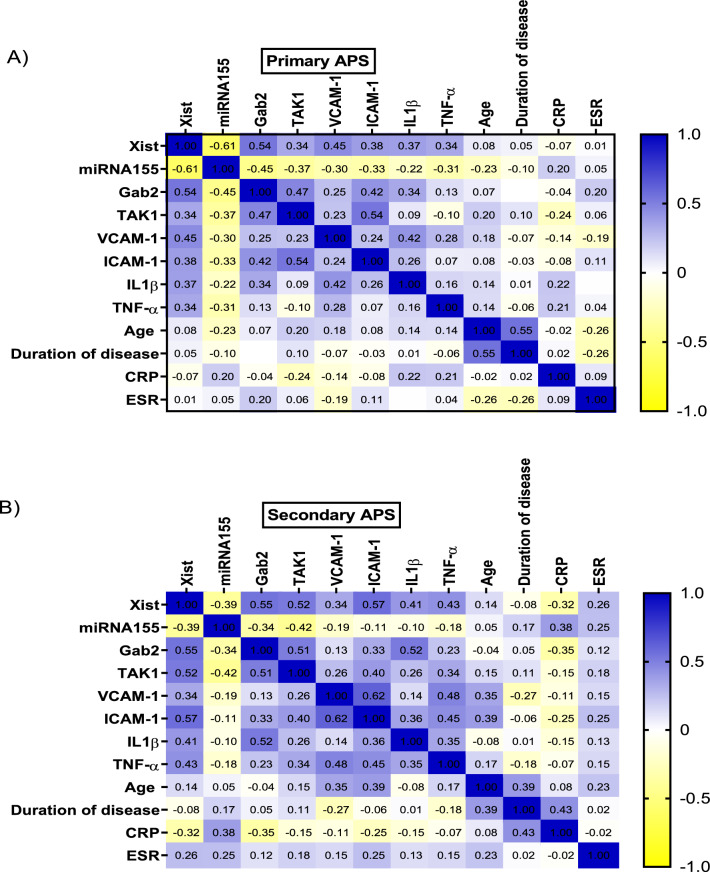
Double gradient heat map graph representing the correlations between different studied parameters in primary (**A**) and secondary (**B**) APS patients. Blue color represents the strong positive correlation (1), while yellow color represents the negative correlation (− 1). Gab2: Grb2-associated binding, ICAM-1: Intracellular cell adhesion molecules, IL-1ꞵ: Interleukin1ꞵ, TAK1: Transforming growth factor beta-activated kinase 1, TNF-α: Tumor necrosis factor-α, VCAM-1: Vascular cell adhesion molecules.

**Figure 5:**
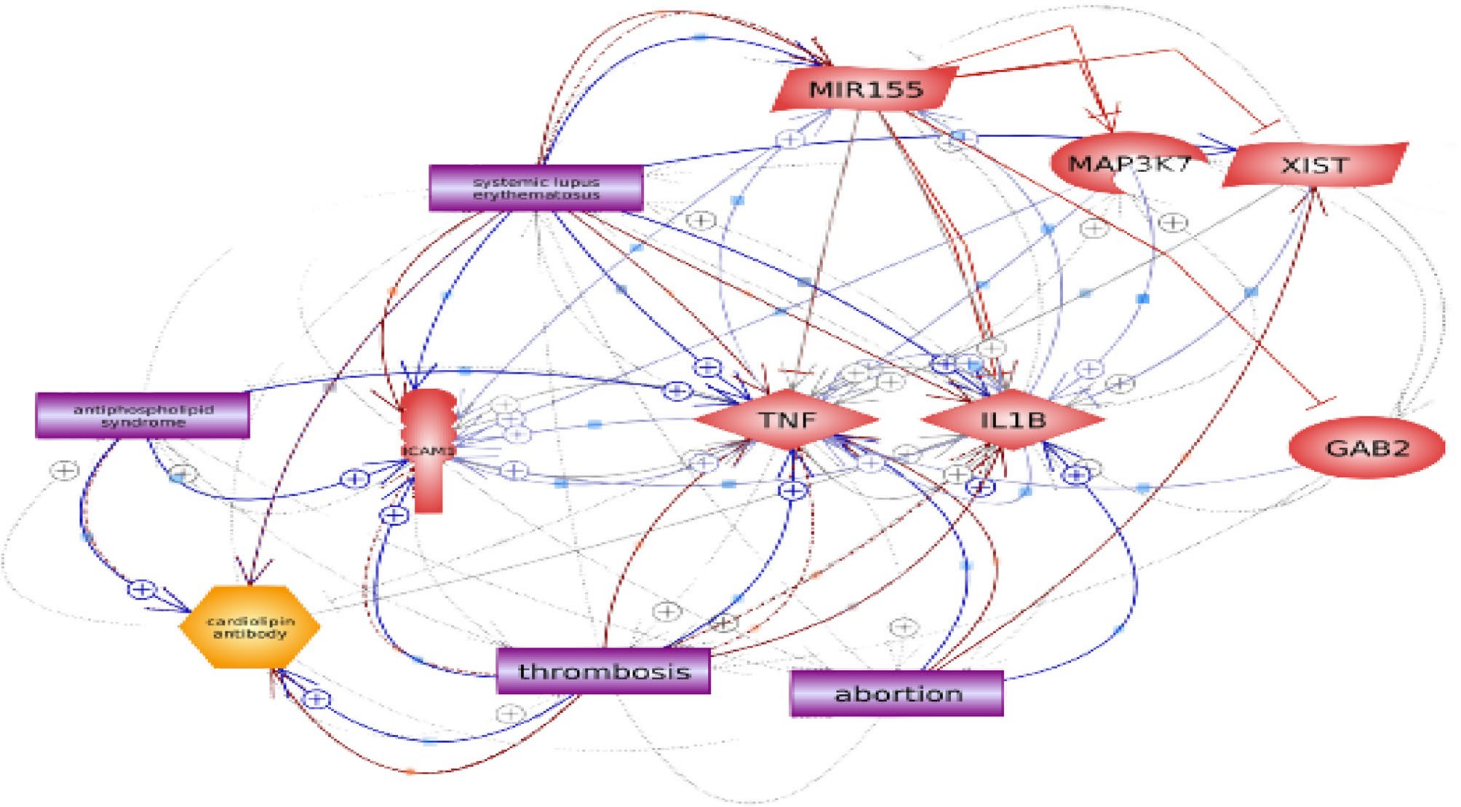
Integrated network representing the interaction between the studied genes. Gab2: Grb2-associated binding, ICAM-1: Intracellular cell adhesion molecules, IL-1ꞵ: Interleukin1ꞵ, MAP3K7 /9TAK1): Transforming growth factor beta-activated kinase 1, TNF-α: Tumor necrosis factor-α, VCAM-1: Vascular cell adhesion molecules.

Among primary APS patients, the lncRNA XIST was directly correlated with the levels of Gab2, TAK1, VCAM-1, ICAM-1, IL-1ꞵ, and TNF-α. In opposition, miRNA155 was reciprocally correlated with the level of Gab2 and TAK1. Likewise, the lncRNA XIST was directly correlated with the level of Gab2, TAK1, VCAM-1, ICAM-1, IL-1ꞵ, and TNF-α among secondary APS. The miRNA155 was only inversely correlated with Gab2 and TAK1 as shown in Fig. 4. The age of the patients and duration of the disease failed to show any significant association with XIST and miRNA155. The interaction between XIST and miRNA155 and other parameters are visualized in Fig. 5 using the Pathway Studio online tool.

### Association of the studied pathway with the occurrence of APS and miscarriage events using logistic regression analysis

The investigated genes and their association with primary and secondary APS were studied using logistic regression analysis. As shown in Table 1, XIST and miRNA155 along with Gab2, TAK1, VCAM-1, ICAM-1, IL-1ꞵ, and TNF-α were identified as the final independent variables associated with APS with *P* values 0.004, 0.01, 0.03, 0.005, < 0.0001 < 0.0001, 0.005 and < 0.0001 respectively. Furthermore, logistic regression analysis revealed that XIST, along with VCAM-1, ICAM-1, IL-1ꞵ, and TNF-α are crucial contributors predisposing to miscarriage events among APS patients as shown in Table 1.

**Table 1:** Logistic regression analysis model to assess the association of the following parameters in antiphospholipid patients.

Variables	B	SE	*P* value	Odds ratio	95% CI
Relationship of the following genes with APS					
XIST	0.77	0.27	0.004*	2.16	1.2–3.6
MiRNA 155	− 0.55	0.23	0.01*	0.57	0.36–0.91
Gab2	0.42	0.2	0.03*	1.53	1.03–2.2
TAK1	0.2	0.1	0.005*	1.34	1.09–1.66
VCAM-1	0.04	0.01	< 0.0001*	1.04	1.02–1.06
ICAM-1	0.33	0.09	< 0.0001*	1.4	1.16–1.68
IL-1ꞵ	0.01	0.006	0.005*	1.01	1.005–1.03
TNF-α	0.03	0.01	< 0.0001*	1.03	1.01–1.05
Relationship of the following genes with miscarriage events					
XIST	0.38	0.12	0.002*	1.46	1.1- 1.86
MiRNA155	− 0.64	0.43	0.14	0.52	0.22–1.24
Gab2	0.41	0.24	0.09	1.5	0.93–2.46
TAK1	0.19	0.1	0.08	1.2	0.97–1.49
VCAM-1	0.04	0.01	0.01*	1.04	1.01–1.07
ICAM-1	0.27	0.11	0.01*	1.32	1.04–1.66
IL-1ꞵ	0.01	0.007	0.03*	1.01	1.01 -1.02
TNF-α	0.03	0.01	0.008*	1.03	1.008–1.05

*Gab2* Grb2-associated binding, *ICAM-1* intracellular cell adhesion molecules, *IL-1ꞵ* Interleukin1ꞵ, *TAK1* transforming growth factor beta-activated kinase 1, *TNF-α* tumor necrosis factor-α, *VCAM-1* vascular cell adhesion molecules.

### Diagnostic power of XIST and miRNA155 in identifying APS and miscarriage events

**Figure 6:**
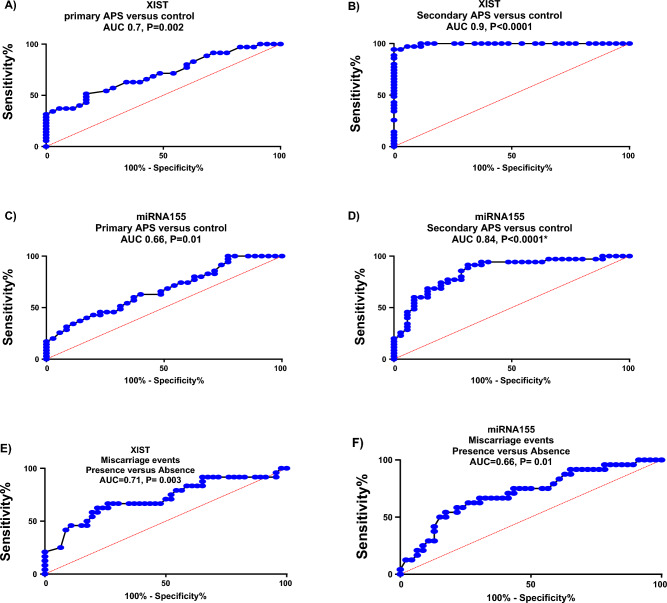
ROC curve to predict the diagnostic performance of XIST and miRNA155 in predicting primary and secondary APS, as well as miscarriage events.

Receiver-operating-characteristic (ROC) curve analysis revealed the accessibility of lncRNA XIST to distinguish between primary and secondary APS from control subjects with AUC = 0.7 and 0.9, *P *= 0.002 and < 0.0001, sensitivity = 63% and 97% and specificity = 66% and 88% at a cutoff = 0.9 and 1.2, respectively. Regarding miRNA155, it can be used in diagnosing primary and secondary APS from control subjects with AUC = 0.66 and 0.84, *P* = 0.01 and < 0.0001, sensitivity = 60% and 80%, specificity = 66% and 72% at a cutoff= 1.05 and 0.8, respectively. Simultaneously, both XIST and miRNA155 can be used in the prediction of miscarriage events with AUC = 0.71 and 0.66, *P* = 0.003 and 0.01, sensitivity = 63% and 66%, specificity = 74% and 67% at a cutoff = 6.3 and 0.29, respectively, as shown in Fig. 6.

### Gene ontology analysis

**Figure 7:**
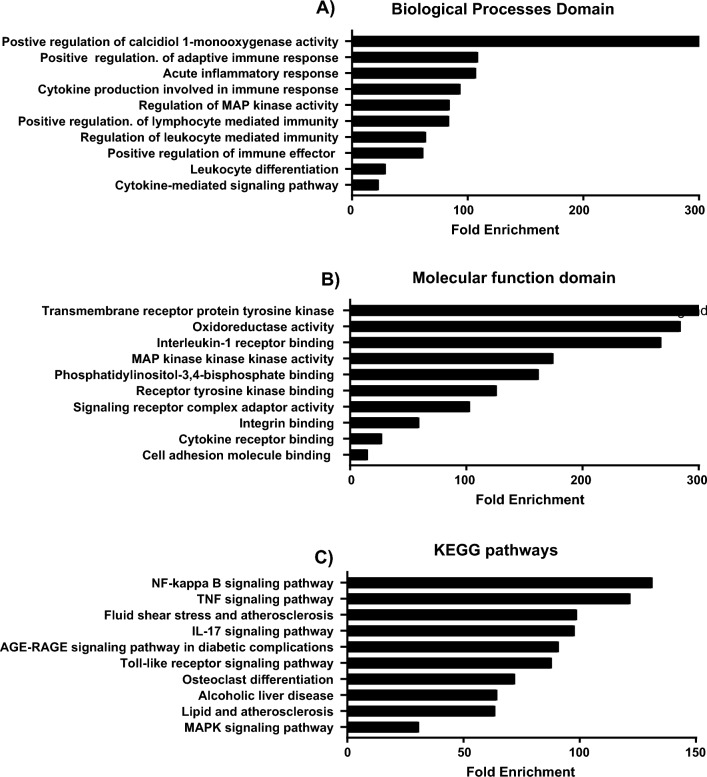
Gene ontology analysis of the examined pathway. (**A**) Biological processes domain, (**B**) Molecular function domain. (**C**) Data relevant to KEGG pathways. Data are sorted based on fold enrichment.

Gene ontology (GO) analysis and Kyoto Encyclopedia of Genes and Genomes (KEGG) were used to identify the participation of the studied genes in different molecular functions and biological as shown in Fig. 7.

### Association of XIST and miRNA155 with other clinic-pathological data of APS patients

Among whole APS patients, the current study conducted stratification analysis to assess the relationship between the formerly studied pathway and different prevailed complications. In supplementary materials Figs. S2 and S3.

## Discussion

The current study is the first report to investigate the role of the lncRNA XIST & miRNA155 in pathophysiology and etiology APS. The current study also investigated the plausible interplay between these non-coding molecules and the Gab2/TAK1 inflammatory cascade as downstream targets and whether this axis affects the thrombotic and obstetrical events associated with APS. Moreover, the study highlighted the interplay of the former genes on VCAM-1, ICAM-1, TNF-α, and IL-1ꞵ. The study went further to assess the validity of the former genes in APS diagnosis and prognosis.

Growing data points towards the putative role of the lncRNA XIST in sex-biased autoimmune diseases^7^. In the present study, the expression level of XIST was highly expressed in primary and secondary APS patients compared with healthy control. Moreover, XIST was dramatically elevated among secondary APS compared with primary ones. This comes consistent with research carried out by Zhang et al. who reported high expression levels of XIST in SLE patients when compared to healthy subjects^6^. These findings may provide genetic evidence for the sex disparity of autoimmune diseases where XIST possesses a vital role in X chromosome inactivation in female cells which is a normally occurring phenomenon during somatic cell differentiation. XIST allows recruiting of multiple proteins that play an important role in X-linked gene silencing. That’s why, XIST owes sex-specific expression patterns and is nominated as an influential gene for sex-biased diseases^6,15^. Interestingly, the present study is the first one focusing on XIST gene expression and APS syndrome either primary or secondary. The currently observed alterations in XIST may underscore the high prevalence of APS and SLE among females more than males. Indeed, preceding epidemiological studies reported that APS is one of the sex-biased diseases where it highly prevails among females, especially in childbearing age^16,17^

Another noncoding RNA studied in the current study is miRNA155; which was significantly downregulated among APS patients compared with healthy control. Moreover, the current study showed marked downregulated miRNA155 expression among secondary patients compared to primary ones. These results are consistent with Pérez-Sánchez et al. who reported a significantly decreased miRNA155 in neutrophils of APS patients^18^. Additionally, Wang et al. revealed the downregulation of miRNA155 among SLE patients^19^. In the opposite direction, Kong et al. declared that the upregulation of miRNA155 suppresses inflammatory cascades among SLE patients via downregulating the ERK pathway^20^. Another study by Thai et al. revealed that miRNA155 promotes the production of autoantibodies where deleting miRNA155 leads to the ablation of deleterious antibodies, and then APS flares are alleviated^21^.

In an attempt to explore the putative regulatory networks that XIST and miRNA155 might recruit to carry out its multifunction, the current study revealed the simultaneous upregulation of XIST with the downregulation of miRNA155. This finding supports the earlier literature that demonstrated that the lncRNAs behave as molecular competitive inhibitors that bind to miRNA and control its functions. Further validation was done via bioinformatics tools that showed the plausible attachment of the seed sequence of the miRNA155 to six different binding sites along the lncRNA XIST. Besides that, previous studies reported the inverse correlation between XIST and miRNA155 in hepatocellular carcinoma^22^,^14^ and cardiac fibroblasts^23^. However, the present study is the first documentation of their role in APS.

Gab2 is a docking protein, that mediates multiple signaling molecules and controls cell autonomy^24^. Yet its role in mediating immune responses wasn’t fully explained. The present study revealed that gene and protein expressions of Gab2 were significantly elevated in the APS groups compared with healthy controls. Indeed previous reports described the potential role of Gab2 in propagating inflammatory and immunological cascades^10^,^25^ which are the main domains of APS pathological mechanism. Interestingly, the current report demonstrated that Gab2 was directly correlated with XIST synchronously with an indirect correlation with miRNA155. These data are in harmony with Xiong et al. who reported that XIST upregulates Gab2 in the cerebral ischemia model^9^. Moreover, Liu et al. reported that the suppression of Gab2 is one of the targets of miRNA155 to mediate its anti-inflammatory role in toxin-induced inflammatory injury^26^. Thus, the present study is the first to postulate the link between both lncRNA XIST & miRNA155 and Gab2 scaffold protein in APS patients.

In the same context, TAK1; the ubiquitin-dependent kinase; is considered an influential enhancer of diverse transcription factors so it is a key player in genetic controlling of inflammatory and immunological processes^27,28^. The present study disclosed the upregulated gene and protein expression levels of TAK1 among APS groups in comparison with healthy control. These data are consistent with the earlier reports which confirmed that TAK1 is a key player in adaptive immunity, where it regulates signaling from T- and B-cell receptors^29^. Moreover, the current study added that TAK1 was positively correlated with XIST and negatively correlated with miRNA155. Despite the paucity of research on the cellular and molecular link between the former noncoding RNAs and TAK1, the majority of reports have recognized TAK1 as a pivotal downstream target for many noncoding RNAs in many diseases. Zheng et al. reported that the lncRNA MIR122HG and the miRNA122-5p modulate innate immunity responses via TAK1^30^. Another report by Li et al. demonstrated that the lncRNA TUG1 stimulates miR-27b-3p and exerts fibrotic activity via activating the TAK1 pathway^31^. Collectively, all of this evidence suggests that XIST and miRNA155 may be the upstream regulators of TAK1, which in turn triggers inflammatory and immunological cascades in autoimmune diseases such as APS. It is noteworthy that the current study demonstrated that Gab2 was directly correlated with TAK1 confirming the currently studied axis; XIST/miRNA155/Gab2/TAK1.

Undoubtedly, there is a straightforward association between APS and CAMs which finally leads to a thrombogenic environment. Likewise, the present study reveals that VCAM-1 and ICAM-1 were markedly elevated among either primary or secondary APS patients in agreement with previous studies^32^. Interestingly, the current study illustrated that the gene expression of the lncRNA XIST was directly correlated with VCAM-1 and ICAM-1 which is coherent with preceding studies that confirmed the role of lncRNA XIST upregulation in atherosclerosis^33,34^. Consequently, the present findings suggest that the lncRNA XIST may underscore at least partially the leukocyte recruitment and adhesion observed among APS patients via stimulating the secretion of VCAM-1 and ICAM-1 which ultimately triggers thrombotic reactions.

The pro-inflammatory cytokines are considered double edged-sword as they guard the host when exposed to any inflammatory stimuli. But other times, extra production of these immunological mediators in autoimmune diseases may endanger the host^35,36^. In the current study, the sera levels of both IL-1ꞵ and TNF-α were highly elevated among APS patients reflecting the ongoing inflammatory state in those patients. Moreover, the lncRNA XIST was positively correlated with IL-1ꞵ and TNF-α consistently with previous studies which disclosed that the knockdown of XIST aids in attenuating the inflammatory response^37,38^. Interestingly, these results may justify the differences in inflammatory responses based on biological sex which make females more prone to inflammatory and immunological disturbances. Thus the involvement of XIST in versatile pathological mechanisms may be the drive beyond various female-predominant disorders^39^. In the opposite direction, the miRNA155 didn’t show any significant role in modulating the levels of CAMs and pro-inflammatory cytokines IL-1ꞵ and TNF-α. These findings may be attributed to the interrelated role of these markers in several intracellular pathways, so maybe fine-tuned by other miRNAs^40–42^.

For more validation of the interplay of the candidate markers in APS, logistic regression analysis was done. The lncRNA XIST and miRNA155 along with Gab2, TAK1, VCAM-1, ICAM-1, IL-1ꞵ, and TNF-α were identified as the final independent variables in APS. Furthermore, the APS patients were stratified based on their obstetrical complications history such as late abortion, early abortion, and premature labor. Logistic regression analysis revealed that XIST, along with VCAM-1, ICAM-1, IL-1ꞵ, and TNF-α are crucial contributors predisposing to miscarriage events among APS patients. Consistently, Guo et al. addressed the upregulated XIST among preeclampsia patients by inhibiting the proliferation and invasion of trophoblastic cells^43^. The upregulation of XIST followed by elevated levels of inflammatory cytokines and CAMs may be the underlying mechanism at least partially of the recurrent abortion events in APS. Noteworthy that pro-inflammatory cytokines such as IL-1ꞵ and TNF-α may promote stickiness of the endothelial cells, platelets, and white cells causing pro-coagulant liability and depressed blood reaching the endometrial tissues^12^,^44^.

In terms of diagnostic performance, ROC curve analyses revealed the predictive power of the lncRNA XIST and miRNA155 to differentiate between APS patients and control subjects. Consequently, they may serve as trustworthy noninvasive biomarkers for the diagnosis of APS and promising therapeutic targets, which may aid in improving the efficiency and accuracy of diagnosis and prognosis of APS. At the same time, the study suggests that both XIST and miRNA155 could be valuable markers for the prediction of miscarriage events; however, they need further clinical investigations. This is in agreement with preceding studies which document the utility of these noncoding genes as noninvasive accurate, and reproducible, diagnostic and prognostic tools^45–47^

Last but not least, the bioinformatics extension of the current study in an attempt to open a new area for scientific research revealed the participation of the studied genes in many biological and molecular tasks such as regulation of leukocyte-mediated immunity, modulation of nuclear factor kappa B activity, T cell cytokine production, controlling interleukin-2 release and T cell-associated cytokine, phosphatidylinositol-3,4-bisphosphate binding, MAP kinases activity, and interleukin-1 receptor binding. However, further clinical studies are needed on a large number of patients as well as should be conducted on other autoimmune diseases.

## Conclusions and prospects

The present study provides for the first time novel insights about the epigenetic aspect of APS and suggests that the noncoding RNAs; lncRNA XIST and miRNA155 could be the upstream regulators of Gab2/TAK1 axis among primary and secondary APS patients. Moreover, the former axis mediates its deleterious effect via modulating the levels of VCAM-1, ICAM-1, IL1ꞵ, and TNF-α which propagates further inflammatory and immunological streams. Interestingly, the study addressed that lncRNA XIST and miRNA155 may be responsible for the thrombotic and miscarriage events associated with APS and provides new non-invasive biomarkers for diagnosing the disease and tracking its progression. Upcoming clinical studies and more practical considerations in this path would lead to a better understanding of the pathological mechanisms of APS which ultimately lead to improving the treatment strategy.

## Subjects and methods

### Subjects

One hundred and five subjects were engaged in the current study. The sample size was determined using the G*power software sample size online calculator (http://www.ghu.de/en/html) based on a two-sided 95% confidence level and a power of 0.90. Seventy patients were diagnosed as APS; thirty-five patients had primary APS & thirty-five patients had APS secondary to SLE with mean ages 30.2 ± 9.7 and 32.5 ± 9.1, respectively. Additionally, age and sex-matched thirty-five control subjects were recruited with a mean age of 30.7 ± 11.7 years. The criteria for selection of the APS study population were based on at least one clinical and one laboratory criterion according to the updated international consensus classification criteria^48^. Primary patients were diagnosed as APS only without any other autoimmune disease, whereas all secondary patients were secondary to SLE. The patients with SLE met at least four of the American College of Rheumatology (ACR) revised criteria for SLE (2019)^49^. Additionally, patients with miscarriage events were categorized based on any history of late abortion, early abortion, and premature labor. Exclusion criteria were coexistance of pregnancy, malignancies, thyroid gland disorders, autoinflammatory disorders and any other autoimmune disease other than APS and SLE.

All the participants enrolled from Rheumatology Immunology Clinics, Internal Medicine, Faculty of Medicine, Cairo University during the period from August 2022 to November 2022. Data were collected through designed questionnaire discussions. Anthropometry, clinical, and laboratory assessments were carried out to rule out any other autoimmune diseases. Whole participants signed informed consent before the donation of blood according to the ethical standard laid down in the Helsinki Declaration and current national laws. All methods were permitted by the Research Ethics Committee of Faculty of Pharmacy, Cairo University, Cairo, Egypt (Permit number: BC 3035) and performed in accordance with their guidelines and regulations.

## Methods

### Peripheral blood sampling

Blood samples were withdrawn from each participant after fasting for eight hours. Blood was collected into EDTA-containing vacuum tubes. Serum samples were separated by a double-centrifugation protocol (3500×*g* for 15 min at 5 °C and 15,000×*g* for 5 min at 5 °C) using a bench top centrifuge and then kept at − 80 °C until further investigations.

### Quantitative real-time reverse transcription-PCR (RT-PCR)

Total RNA was extracted from serum using TRIzol lysis reagent (Direct-zol, Zymo research Cat# R2052) according to the manufacturing protocol. DNase was included in the kit to assure the purity of the extracted RNA. NanoDrop 2000 spectrophotometer (Thermo Scientific, USA) was used to determine RNA concentration and purity. The 2 μg of the extracted total RNA was used as the template for complementary DNA synthesis which was carried out by COSMO cDNA synthesis kit (Willowfort, UK) (CAT# WF10205001) and performed quantitative PCR in a Real-Time PCR System (Applied Biosystems, USA) using HERA SYBR^®^ Green kit (Willowfort, UK) (CAT# WF1030300X). The expressions of lncRNA XIST, miRNA155, Gab2, and TAK1 were normalized to the level of GAPDH for lncRNA & mRNAs and U6 for miRNA. The primer sequences are listed in Table S2 (Supplementary section). The results were calculated using the 2^−ΔΔCT^ formula previously described by Schmittgen and Livak (2008)^50^.

### Enzyme-Linked ImmunoSorbent assay for determination of VCAM-1, ICAM-1, IL-1ꞵ and TNF-α

In vitro, quantitative determination of serum levels of VCAM-1 and ICAM-1 were measured by commercial ELISA kits which were supplied by MyBioSource (UK). The serum levels of IL-1ꞵ and TNF-α were assessed by using quantitative sandwich ELISA kits supplied by MyBioSource (UK). All the procedures were done as per the manufacturing instructions.

### Western blot analysis for determination of Gab2 and TAK1

Western blotting was used to detect Gab2 and TAK1 in the given sample. The separated proteins were blotted onto a polyvinylidene difluoride membrane where they were incubated with primary antibodies against Gab2 and TAK1. After that, membranes were incubated with a secondary horseradish peroxidase-conjugated antibody. Beta‐actin was employed as the internal control. The chemiluminescent substrate was applied to the blot, and the bands’ intensity was read after normalization to β‐actin on a Chemi Doc MP imager (BIO‐RAD).

### Bioinformatics analysis

Bioinformatics database analysis was done for comprehensive exploring of the pathway enrichment analysis as well as gene ontology for the candidate genes. Likewise, the KEGG database was used to analyze the prospective rules of these target genes in these pathways http://bioinformatics.sdstate.edu/go/^51–53^ Additionally, interactions between lncRNA XIST and miRNA155 were examined (http://www.mircode.org & http://bioinfo.life.hust.edu.cn/lncRNASNP). Furthermore, the overlapping among these enriched pathways was visualized using interactive integrated networks.

### Statistical methods

Data were analyzed using GraphPad Prism 9.0 statistics software (San Diego, CA, USA). The normality of data was checked using Kolmogorov–Smirnov and Shapiro–Wilk tests. Parametric data were represented as mean ± SD and tested using One way ANOVA test followed by Tukey’s test. Non-parametric data were represented as median (25–75%) and analyzed using Mann–Whitney U test and Kruskal–Wallis test followed by Dunn’s test for multiple comparisons. Categorical data were expressed as numbers (%) and analyzed using the chi-square (X^2^) test. Spearman’s rho correlation and logistic regression analysis were done to evaluate the association between the measurements. The ROC curve was used to evaluate the studied parameters’ diagnostic accuracy, and the area under the curve (AUC) was determined. The *p*-value was considered statistically significant at *p* equal to or less than 0.05.

### Supplementary Information

Supplementary Information.

## Data Availability

The data that support the study are available from the corresponding author upon reasonable request.
