# Supplementary material for: Association of XIST/miRNA155/Gab2/TAK1 cascade with the pathogenesis of anti-phospholipid syndrome and its effect on cell adhesion molecules and inflammatory mediators
Source: Sci Rep. 2023 Nov 1;13:18790. doi: 10.1038/s41598-023-45214-z (PMC10620142; doi:10.1038/s41598-023-45214-z)
Supplement: Supplementary file 1 — Supplementary Information. [file 41598_2023_45214_MOESM1_ESM.docx]

**Table S1: Demographic and clinical data of the studied groups.**

| **Variables** | **Primary APS**  **(n=35)** | **Secondary APS**  **(n=35)** | **Control subjects**  **(n=35)** | **P value** |
| --- | --- | --- | --- | --- |
| **Age (Years)** | **30.2 ± 9.7** | **32.5 ± 9.1** | **30.7 ± 11.7** | **0.61** |
| **Gender, Female** | **32 (91.4%)** | **33 (94.2%)** | **33 (94.2%)** | **0.85** |
| **Weight (kg)** | **73.5 ± 9.2** | **70.8 ± 10.6** | **71.3 ± 10.4** | **0.49** |
| **Duration of disease (months)** | **20.4 ± 15.8** | **26.8 ± 18.4** | **-** | **0.12** |
| **Positive Anticardiolipin IgM** | **23 (65.7%)** | **24 (68.5%)** | **-** | **0.7** |
| **Anticardiolipin IgM (U/ml)** | **26.4 ± 22.3** | **22.1 ± 17.3** | **-** | **0.37** |
| **Positive Anticardiolipin IgG** | **21 (60%)** | **19 (54.2%)** | **-** | **0.19** |
| **Anticardiolipin IgG (U/ml)** | **34.5 ± 30.6** | **30.1 ± 29.3** | **-** | **0.52** |
| **Positive Lupus anticoagulant IgG** | **20 (57.1%)** | **22 (62.8%)** | **-** | **0.62** |
| **Lupus anticoagulant IgG (U/ml)** | **47.8 ± 22.6** | **46.5 ± 26.7** | **-** | **0.82** |
| **Positive Anti Beta2 glycoprotein IgM** | **17 (48.5%)** | **19 (54.2%)** | **-** | **0.63** |
| **Anti Beta2 glycoprotein IgM (U/ml)** | **24.4 ± 12.8** | **23.4 ± 12.4** | **-** | **0.73** |
| **Positive Anti Beta2 glycoprotein IgG** | **10 (31.2%)** | **14 (42.4%)** | **-** | **0.31** |
| **Anti Beta2 glycoprotein IgG (U/ml)** | **27.9 ± 26.5** | **25.2 ± 23.1** |  | **0.64** |
| **Anti-nuclear protein** | **-** | **33 (94.2%)** | **-** | **-** |
| **Anti-ds-DNA** | **-** | **34 (97.1%)** | **-** | **-** |
| **Triple positivity** | **6 (17.1%)** | **12 (34.2%)** | **-** | **0.1** |
| **Early abortion** | **12 (37.5%)** | **13 (39.3%)** | **-** | **0.8** |
| **Late abortion** | **10 (31.2%)** | **14 (42.4%)** | **-** | **0.31** |
| **Premature labor** | **12 (37.5%)** | **20 (60.6%)** | **-** | **0.05** |
| **Deep venous thrombosis (DVT)** | **13 (37.1%)** | **14 (40%)** | **-** | **0.8** |
| **Superficial thrombophlebitis** | **4 (11.4%)** | **3 (8.5%)** | **-** | **0.69** |
| **Pulmonary embolism** | **11 (31.4%)** | **9 (25.7%)** | **-** | **0.59** |
| **Myocardial infarction** | **8 (22.8%)** | **7 (20%)** | **-** | **0.87** |
| **Transient ischemic attacks** | **6 (17.1%)** | **4 (11.4%)** | **-** | **0.49** |
| **Stroke** | **12 (34.2%)** | **9 (25.7%)** | **-** | **0.43** |
| **Budd-Chiari** | **4 (11.4%)** | **2 (5.7%)** | **-** | **0.39** |
| **Avascular necrosis** | **7 (20%)** | **12 (34.2%)** | **-** | **0.17** |
| **Livedo reticularis** | **17 (48.5%)** | **15 (42.8%)** | **-** | **0.63** |
| **Ischemia/ gangrene** | **5 (14.2%)** | **6 (17.1%)** | **-** | **0.74** |
| **Hemolytic anemia** | **10 (28.5%)** | **10 (28.5%)** | **-** | **1** |
| **Thrombocytopenia** | **17 (48.5%)** | **20 (57.1%)** | **-** | **0.47** |
| **Proteinuria** | **4 (11.4%)** | **24 (68.5%)** | **-** | **<0.0001** |

Data are represented as mean ± SD or Number (%). The percentages of early abortion, late abortion, premature labor pateints were calculated in females only. Statistical differences between the two groups were calculated using independent student t-test or chi-square test (X^2^). Statistical differences between the three groups were calculated using One Way ANOVA or chi-square test (X^2^).

**Table S2: List of primers used**

| **Gene** |  | **Sequence** |
| --- | --- | --- |
| **LncRNA XIST** | Forward | 5’-AGCTCCTCGGACAGCTGTAA-3’ |
|  | Reverse | 5’-CTCCAGATAGCTGGCAACC-3’ |
| **miRNA155** | Forward | 5'-CTCGCTTCGGCAGCACA-3' |
|  | Reverse | 5'-AACGCTTCACGAATTTGCG T-3’ |
| **Gab2** | Forward | 5’-CGAAGAGAACTATGTCCCTATGC-3’ |
|  | Reverse | 5’-AGGGGCAGGACTGTTCGT-3’ |
| **TAK1** | Forward | 5’-GCGTCGGAAACCCTTTGA-3’ |
|  | Reverse | 5’-TGAACAGCCCACATGATTCG-3’ |
| **U6** | Forward | 5’-GCTTCGGCAGCACATATACTAAAAT-3’ |
|  | Reverse | 5’-CGCTTCACGAATTTGCGTGTCAT-3’ |
| **GAPDH** | Forward | 5’-CCTGACCTGCGTGTGGACT-3’ |
|  | Reverse | 5’-GCTGTGGATGGGGAGGTGTC-3’ |


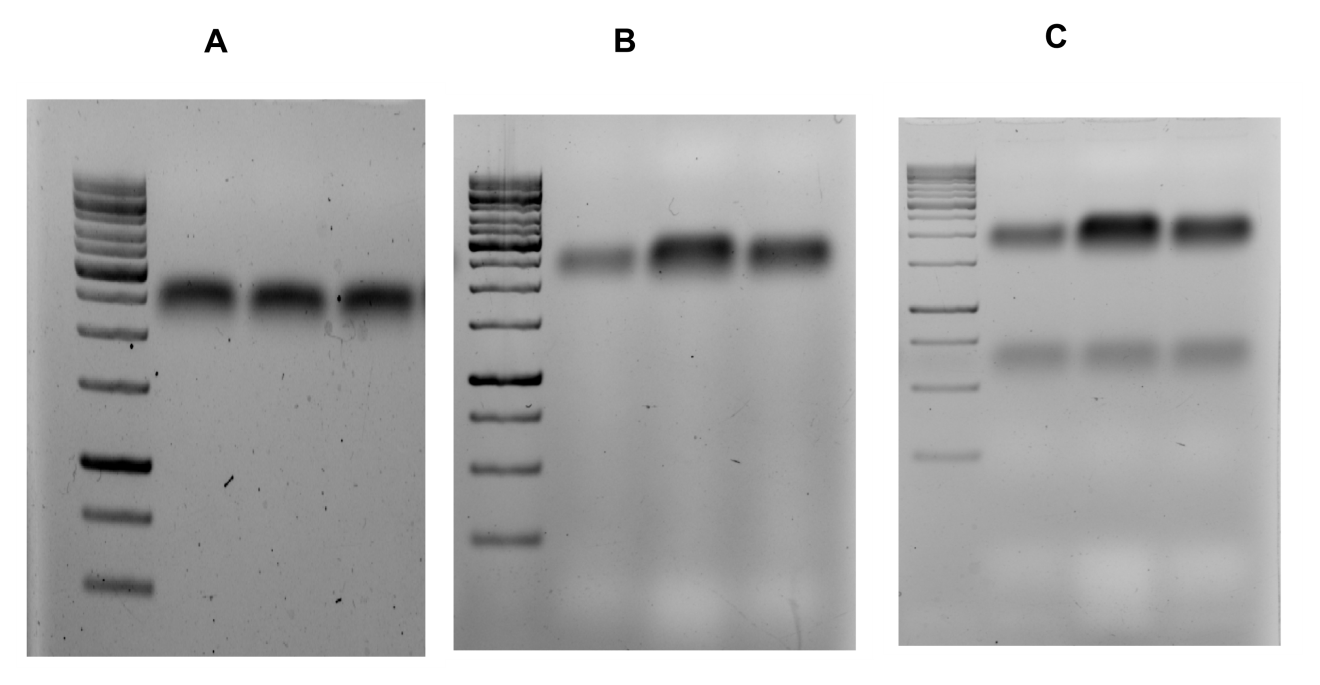


**Figure S1: Un cropped western pictures of B-actin (A), GAB2 (B), and TAK1 (C)**

**Figure S2: Comparison of gene expression profiles of lncRNA XIST and miRNA155 among APS patients with different clinicopathological findings. The box represents the 25–75% percentiles; the line inside the box represents the median and the error bars representing the 10%-90% percentiles.**

**Figure S3: Comparison of gene expression profiles of lncRNA XIST and miRNA155 among APS patients with different clinicopathological findings. The box represents the 25–75% percentiles; the line inside the box represents the median and the error bars representing the 10%-90% percentiles.**
